# Semantic Interpretation for Convolutional Neural Networks: What Makes a Cat a Cat?

**DOI:** 10.1002/advs.202204723

**Published:** 2022-10-10

**Authors:** Hao Xu, Yuntian Chen, Dongxiao Zhang

**Affiliations:** ^1^ BIC‐ESAT ERE and SKLTCS College of Engineering Peking University Beijing 100871 P. R. China; ^2^ Eastern Institute for Advanced Study Yongriver Institute of Technology Ningbo Zhejiang 315200 P. R. China; ^3^ National Center for Applied Mathematics Shenzhen (NCAMS) Southern University of Science and Technology Shenzhen Guangdong 518055 P. R. China; ^4^ Department of Mathematics and Theories Peng Cheng Laboratory Shenzhen Guangdong 518000 P. R. China

**Keywords:** convolutional neural network, interpretable machine learning, semantic space, trustworthiness assessment

## Abstract

The interpretability of deep neural networks has attracted increasing attention in recent years, and several methods have been created to interpret the “black box” model. Fundamental limitations remain, however, that impede the pace of understanding the networks, especially the extraction of understandable semantic space. In this work, the framework of semantic explainable artificial intelligence (S‐XAI) is introduced, which utilizes a sample compression method based on the distinctive row‐centered principal component analysis (PCA) that is different from the conventional column‐centered PCA to obtain common traits of samples from the convolutional neural network (CNN), and extracts understandable semantic spaces on the basis of discovered semantically sensitive neurons and visualization techniques. Statistical interpretation of the semantic space is also provided, and the concept of semantic probability is proposed. The experimental results demonstrate that S‐XAI is effective in providing a semantic interpretation for the CNN, and offers broad usage, including trustworthiness assessment and semantic sample searching.

## Graphical abstract

The semantic explainable artificial intelligence (S‐XAI) proposed in this work can extract common traits via the row‐centered sample compression method and obtain semantically sensitive neurons for visualizing the semantic space. The S‐XAI is effective in providing a semantic interpretation for the convolutional neural network, and offers broad usage, including trustworthiness assessment and semantic sample searching.

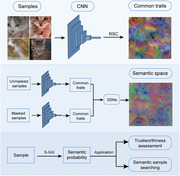

## 1 Introduction

Convolutional neural networks (CNNs) have made tremendous progress in various fields, including computer vision,^[^ ^1^ ^]^ natural language processing,^[^ ^2^ ^]^ and other fields^[^ ^3^ ^]^ in recent years. Although CNNs have achieved superior performance on varied tasks, the degree of confidence for the prediction is limited by its essence as a “black‐box” model, which means that the decision process is difficult to express with explicit rules, and thus challenging to be understood by humans. This shortcoming will expose the CNN to the risk of being attacked or biased.^[^ ^4^ ^]^ Therefore, the interpretability of the CNN has attracted increasing attention, and a variety of techniques have been attempted to explore the decision logic of CNNs in a human‐understandable manner.

Interpretability is defined as the ability to provide explanations in understandable terms to a human.^[^ ^5^ ^]^ Mainstream ways of interpreting CNNs include: 1) feature visualization, which visualizes specific filters or feature maps to depict the representation of a CNN^[^ ^6^, ^7^, ^8^, ^9^, ^10^, ^11^ ^]^; 2) network diagnosis, which diagnoses a pretrained CNN to understand different aspects of it^[^ ^12^, ^13^, ^14^, ^15^, ^16^ ^]^; and 3) structure modification, which adjusts the structure of CNN for better interpretability.^[^ ^17^, ^18^, ^19^, ^20^, ^21^ ^]^ Despite their success, current interpretation methods face fundamental constraints that limit their usage. First, although some studies have focused on semantic information hidden behind the CNN,^[^ ^16^, ^19^, ^22^ ^]^ an explicit understandable semantic space has not yet been abstracted. Second, current researches are centered on extracting individual traits from each sample to obtain local interpretability, and are unable to decipher underlying common traits hidden in the data of the same class. Finally, even though existing works have provided numerous techniques to interpret CNN, few of them inspire applications or improvements in practical tasks. Overall, the study of the interpretation and application of semantic spaces in CNNs still needs to be further developed.

**Figure 1:**
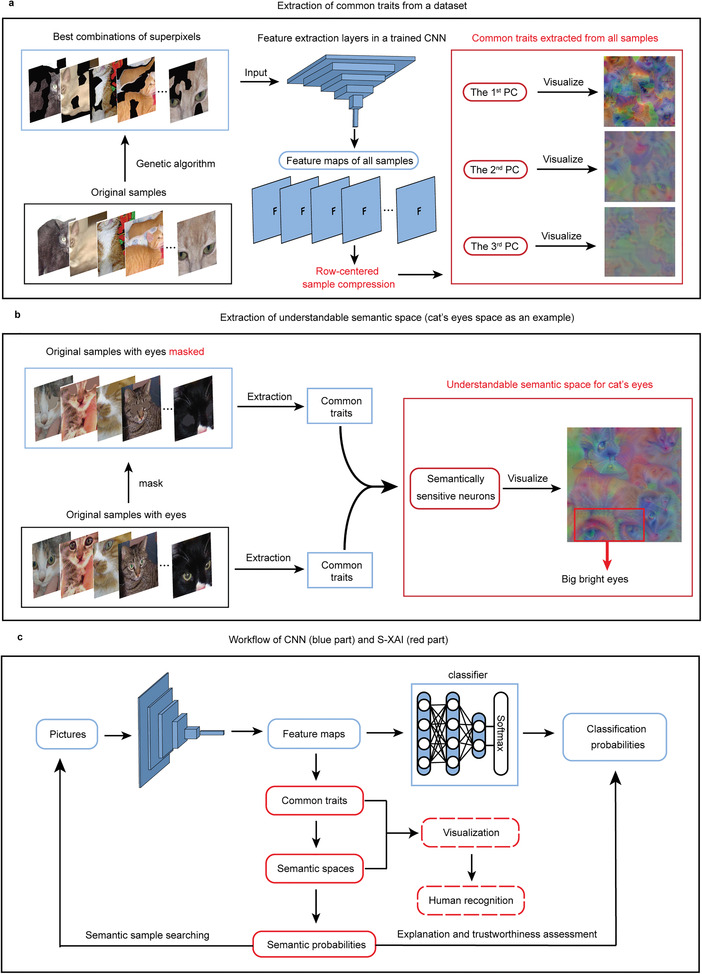
Overview of the proposed S‐XAI. a) Our framework for extracting common traits from a dataset, taking the category of cats as an example. Left: the original samples and discovered best combinations of superpixels. Middle: extracting feature maps for all samples from a pretrained CNN. Right: the obtained principal components (PCs) from the row‐centered sample compression on the feature maps and visualization of common traits. b) Our framework for extracting understandable semantic space, taking the semantic space of cats’ eyes as an example. Left: samples with unmasked and masked semantic concept. Middle: extraction of common traits for both kinds of samples. Right: discovered semantically sensitive neurons (SSNs) and the visualization of the semantic space. The big bright eyes are vividly illustrated, which proves that an understandable semantic space is found. c) The workflow of CNN and S‐XAI. The blue part is the prediction process of the CNN. The red part is the process of S‐XAI, in which the semantic probabilities are calculated from the extracted semantic spaces that can be visualized and recognized by humans for trustworthiness assessment and semantic sample searching. The dashed box refers to an optional step.

In order to overcome these existing limitations, this paper presents the semantic explainable artificial intelligence (S‐XAI), which is a semantic interpreter that provides a global interpretation by abstracting common traits from datasets and extracting explicit understandable semantic spaces from CNNs. It makes the following three notable improvements:

1. Global interpretation via common traits. Instead of seeking the information learned by CNNs in a single image, we adopt a sample compression method based on the distinctive row‐centered principal component analysis (PCA), called row‐centered sample compression (RSC), to explore the common traits hidden behind samples that can be visualized by feature visualization techniques, which is illustrated in **Figure** **1**a.
2. Understandable semantic space. In this work, we extract understandable semantic spaces that can be visualized explicitly, which is shown in Figure 1b. We also propose the concept of semantic probability for the first time, which can measure the probability of occurrence of semantic concepts.
3. Broad and efficient usage. The ultimate goal of understanding neural networks is to elucidate how they work and improve them. Our proposed S‐XAI is able to handle more tasks efficiently, such as trustworthiness assessment and semantic sample searching, thus improving the confidence and scalability of CNNs, which is illustrated in Figure 1c.

In this work, we take the task of discriminating between cats and dogs using the commonly used VGG‐19 network^[^ ^23^ ^]^ as an example to demonstrate what makes a cat recognized as a cat for CNN from the perspective of semantic space. In the VGG‐19 network, we add a global average pooling (GAP) layer before the fully‐connected layer. The GAP layer reduces dimension, and preserves the spatial information extracted by the previous convolutional layers and pooling layers, which facilitates feature visualization.^[^ ^24^ ^]^ In this paper, we first exhibit how to obtain underlying common traits, which are vectors containing mixed semantic features. Then, we describe how to extract understandable semantic space on the basis of the visualization of semantically sensitive neurons (SSNs) discovered from the comparison between the common traits of samples with masked and unmasked semantic concepts. Finally, we introduce the concept of semantic probability and discuss some major challenges for CNNs, including overconfidence for prediction^[^ ^25^ ^]^ and target sample searching,^[^ ^26^ ^]^ and show how the proposed S‐XAI can handle these issues in a simple yet effective way through several experiments.

## 2 Results

### 2.1 Extraction of Common Traits from Samples

**Figure 2:**
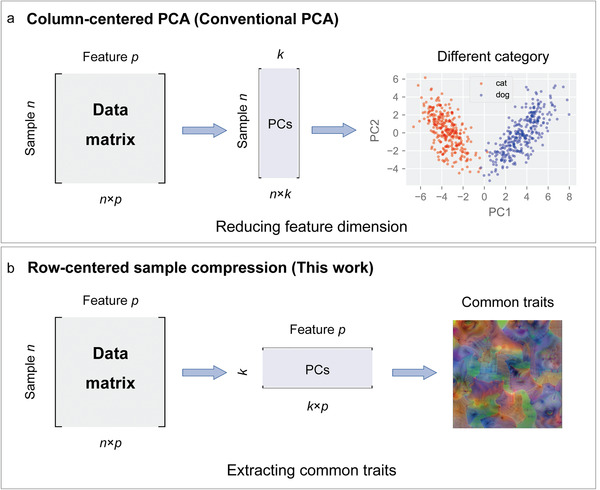
The difference between the conventional column‐centered PCA and the proposed row‐centered sample compression. a) The column‐centered PCA is the conventional PCA commonly used in previous feature visualization methods. b) The row‐centered sample compression based on the distinctive row‐centered PCA is utilized in this work to extract common traits.

In nature, the same kinds of objects often possess certain similar common characteristics, which are termed “common traits.” These common traits constitute an important basis for the identification of species. For example, although there are numerous kinds of cats in the world, they all have similar faces, noses, and legs that are common traits for recognition as cats by humans. In previous literature, some techniques have been proposed to interpret CNNs by finding the part of an image with the largest response to the specified category^[^ ^7^, ^12^ ^]^; however, this is a local interpretation since it only explains why one simple image is judged as a responsive label by the CNN. In other words, these studies have discovered individual traits, instead of common traits. In this work, we innovatively introduce a RSC method to extract common traits from samples for CNNs, which is based on the distinctive row‐centered PCA. **Figure** **2** demonstrates the difference between our proposed RSC method and the conventional PCA. From the figure, the difference can be seen vividly. For the conventional column‐centered PCA, the PCA is conducted to reduce the dimension of features from *p* to *k* (*k* < *p*), which helps to map the high‐dimensional data to low‐dimensional space for better visualizing features of different categories^[^ ^27^ ^]^ (Figure 2a). Therefore, the data matrix is column‐centered to calculate the covariance matrix, which is why the conventional PCA is also called the column‐centered PCA. In contrast, in the proposed RSC method, the data matrix is row‐centered to obtain a different covariance matrix in order to reduce the sample dimension from *n* to *k* (*k* < *n*), which is rarely utilized but is highly appropriate for extracting common traits of samples (Figure 2b). Considering that the feature dimension remains the same during the RSC, it is especially suitable for visualizing and interpreting the CNN.

**Figure 3:**
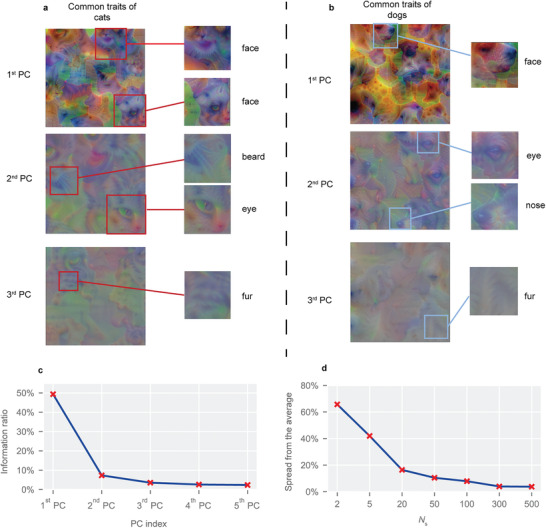
The results for extracting common traits. a) Visualization of the 1st, 2nd, and 3rd PCs for cats (*N*_s_ = 300). The left pictures are original visualized common traits, and the right ones are partial enlarged pictures with explicit traits for better recognition. b) Visualization of the 1st, 2nd, and 3rd PCs for dogs (*N*_s_ = 300). The left pictures are original visualized common traits, and the right ones are partial enlarged pictures. c) The information ratio of the first five PCs extracted from 500 dog samples. The x‐shape annotation refers to the information ratio. A higher information ratio means more feature information contained in the PC. d) The spread from the average for the 1st PC of cats with different *N*_s_. A lower spread means a higher stability of extracted common traits.

The framework for extracting common traits from samples is illustrated in Figure 1a. In the framework, *N*_s_ different cat samples are randomly selected from the dataset, and a specific genetic algorithm is utilized to obtain the optimal combinations of superpixels for each sample, which aims to reduce interference and makes the extracted common traits more representative (Supporting Information S1). The discovered combinations of superpixels are fed into the CNN, and the feature maps are generated after the global average pooling (GAP) layer. Different from previous works that visualize feature maps for each sample,^[^ ^6^, ^7^ ^]^ we conduct the RSC on the feature maps of all *N*_s_ samples. Visualization of the 1st, 2nd, and 3rd principal components (PCs) after the RSC with 300 samples for cats and dogs is illustrated in **Figure** **3**a,b, respectively. It is obvious that different PCs present traits at different levels. The 1st PC displays vivid faces, the 2nd PC displays several body regions, such as beards, eyes, and noses, and the 3rd PC mainly presents fur‐like patterns. Different from the visualization of feature maps for one sample, the visualization of PCs contains multiple traits that clearly belong to different samples, which indicates that the PC integrates the common traits from the samples. The information ratios (calculated by the proportion of total variance) of the first five PCs are presented in Figure 3c, which reveals that the 1st PC retains nearly 50% of the information, while the 2nd PC and 3rd PC retain only 7.32% and 3.53% of the information, respectively. This may explain why the 1st PC can display faces, which are a holistic concept, while the others can only present fragmental semantic parts. Considering the dominant information ratio of the 1st PC, the subsequent study will extract semantic spaces based on the 1st PC.

In order to further prove the stability of the extracted common traits, we conduct repeated experiments with different numbers of samples and calculate the spread from the average, which is expressed as

(1) $$e=\frac{1}{p}\frac{1}{N_{e}}\sum_{j=1}^{p}\sum_{i=1}^{N_{e}}\left|s_{j}^{i}-{\bar{s}}_{j}\right|\times 100\%$$

where *N*_e_ is the number of repeated experiments; *p* is the number of features (i.e., the length of the PC); $S_{j}^{i}$ is the value of the *j*th index in the scores of PCs in the *i*th experiment; and ${\bar{S}}_{j}$ is the average value of the *j*th index in the scores of PC of all of the experiments, which is given as follows

(2) $${\bar{s}}_{j}=\frac{1}{N_{e}}\sum_{i=1}^{N_{e}}s_{j}^{i}$$

In this work, *N*_e_ is 3 and *p* is 512. The result is shown in Figure 3d. From the figure, it is found that the spread tends to decrease as the number of selected samples *N*_s_ increases, which indicates that the extracted traits tend to be more stable with more *N*_s_. Specifically, when the *N*_s_ is 300, the spread is only 3.9%, which means that we can extract stable common traits from a small number of samples, which further proves that certain representative common traits indeed exist in the samples of the same class.

### 2.2 Extraction and Visualization of Understandable Semantic Space

**Figure 4:**
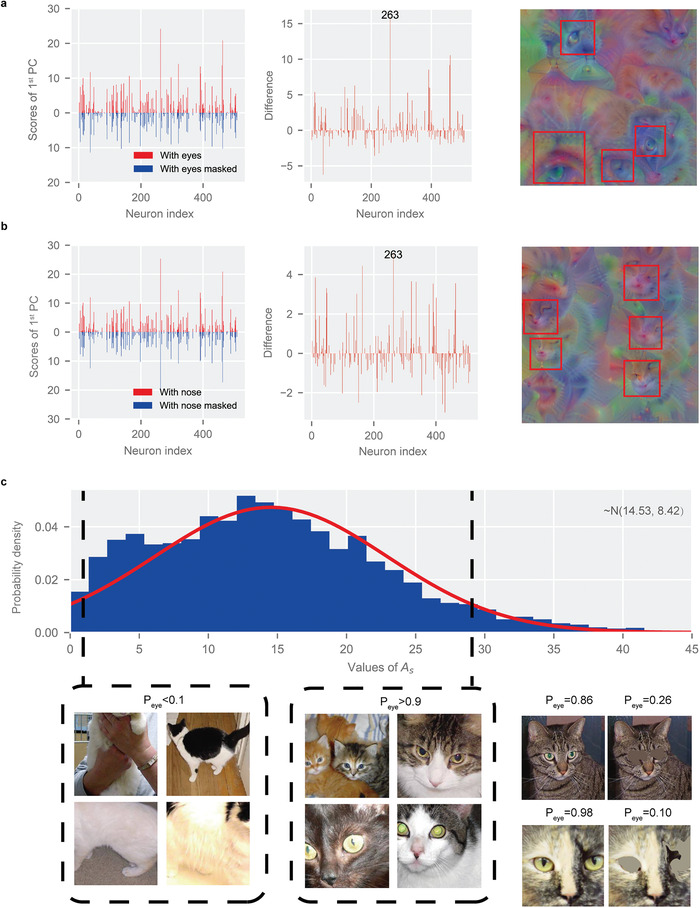
Results of extracted understandable semantic space. a) Left: scores of the 1st PC (i.e., common traits) of samples with eyes unmasked and masked. Middle: difference between the extracted common traits. Right: visualization of extracted semantic space of cats’ eyes, and the red frames are recognized eyes. b) Left: scores of the 1st PC of samples with nose unmasked and masked. Middle: difference between the extracted common traits. Right: visualization of extracted semantic space of cats’ noses, and the red frames are recognized noses. The annotation “263” in both a) and b) indicates that the same SSN occurs in both semantic spaces. c) Probability density distribution plot of the values of the weighted average activation *A*_s_ for 3000 samples of cats. The red curve is the fitted normal distribution curve. The pictures in the black framed lines are the samples located at the left (*P*_eye_ < 0.1) and right (*P*_eye_ >0.9) ends of the distribution, respectively. Bottom right: the semantic probability of samples with eyes unmasked and masked in the semantic space of cats’ eyes.

Although the common traits have been extracted successfully, the semantic information is disorganized since many kinds of semantic concepts are mixed in the extracted common traits. Therefore, we develop a framework to extract and visualize understandable semantic spaces on the basis of the common traits, which is illustrated in Figure 1b. The core of our proposed framework is to compare the common traits extracted from samples with masked and unmasked semantic concepts. We take the semantic space of cats’ eyes as an example. First, *N*_s_ cat samples with visible eyes are chosen from the datasets, and their eyes are masked with surrounding color. Then, the respective common traits are extracted, as shown in **Figure** **4**a. A number of semantically sensitive neurons (SSNs), *N*_SSN_, are chosen by selecting the maximum absolute difference between both common traits, which are visualized in Figure 4a,b, respectively. The semantic concept is split successfully since there only exist multiple bright and clear eyes in the semantic space of cats’ eyes, and the condition is the same for the semantic space of cats’ noses. It is worth noting that the visualized eyes and noses are surreal, i.e., they are not obtained by visualizing a single sample, but rather by concretizing the entire semantic space. The results show that the representation of features at the semantic level from the CNN is successfully obtained.

In this work, the semantic concept is defined by humans to be better understood. However, in our research, we found that the connections between these semantic concepts seem to be more complex. For instance, the 263th neuron is sensitive to both cats’ eyes and nose, which suggests that the network seems to learn the relationship between cats’ eyes and nose. This is also proven by the fact that a blurry nose also appears in the semantic space of cats’ eyes. This means that the semantic concepts learned by the network may be somewhat different from the semantic concepts recognized by humans, which calls for deeper investigation in future work.

### 2.3 Statistical Interpretation for Semantic Space

In order to explore semantic space more deeply, we also provide statistical interpretation for semantic space. For the purpose of depicting the activation extent for an image *z* in the semantic space *s*, we define a weighted average activation *A*_s_(*z*) as

(3) $$A_{s}\left(z\right)=\frac{1}{N_{\mathrm{SSN}}}\sum_{i=1}^{N_{\mathrm{SSN}}}a_{i}\Delta_{i},$$

where *N*_SSN_ is the number of discovered SSNs; *a_i_* is the activation of the *i*th SSN in the feature map extracted from the GAP layer before fully‐connected layers for image *z*; and Δ*_i_* is the value of difference for the *i*th SSN obtained from semantic space. A larger *A*_s_ means that the image has a larger activation in semantic space *s*. Since the calculation process is very fast, the weighted average activation of a large number of samples can be quickly obtained. In this work, we randomly selected 3000 samples containing a wide variety of cats from the dataset and calculated their weighted average activation in the extracted semantic space of cats’ eyes. The probability density distribution plot of the values of the weighted average activation *A*_s_ is displayed in Figure 4c. The distribution is close to a normal distribution, which also occurs in other semantic spaces (Supporting Information S3). Furthermore, we find that the two ends of the probability density distribution correspond to cats without and with obvious eyes, respectively (see Figure 4c). While images of cats without visible eyes are still able to be identified as cats with a high probability by the CNN, their activations within the semantic space of cats’ eyes are low, which further confirms the validity of the extracted semantic space.

In order to measure the relative position of image *z* in the distribution of samples in semantic space *s*, we propose the concept of semantic probability P*_s_* (*z*), which is written as follows

(4) $$P_{s}\left(z\right)=\frac{cdf\left(X=A_{s}\left(z\right)\right)-cdf\left(X=A_{\min}\right)}{cdf\left(X=A_{\max}\right)-cdf\left(X=A_{\min}\right)}$$

where *cdf* is the cumulative distribution function of the fitted normal distribution; and *A*_min_ and *A*_max_ are the min and max of *A*_s_ in the distribution, respectively. A larger P*_s_* (*z*) represents a greater activation of image *z* in the corresponding semantic space *s* with a higher probability of occurrence. Unlike the probability in vector space that usually occurs in CNN studies, the P*_s_* (*z*) here represents the semantic probability in the semantic space, which can measure the probability of a specific semantic concept appearing in the picture. From the figure, the P_eye_(*z*) of the picture with masked eyes exhibits a marked decrease compared with the same one with unmasked eyes in the semantic space of cats’ eyes. Similarly, the images satisfying P_eye_(*z*)>0.9 have large bright eyes, while the images satisfying P_eye_(*z*)<0.1 hardly show any eyes.

### 2.4 Application: Trustworthiness Assessment

**Figure 5:**
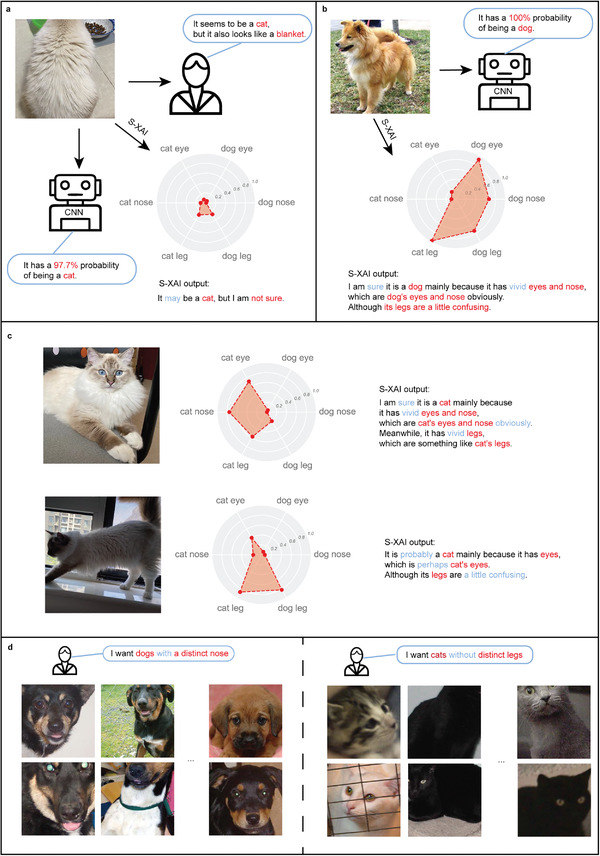
The application of S‐XAI, including trustworthiness assessment and semantic sample searching. a) Assessments given by humans (right), CNN (bottom), and S‐XAI (bottom right) when identifying the picture of a Ragdoll cat facing backward. The assessment of S‐XAI includes a radar map of semantic probabilities in different semantic spaces and an explanation sentence generated automatically from the semantic probabilities. b) Assessments given by CNN (right) and S‐XAI (bottom) when identifying the picture of a dog. c) Assessments given by S‐XAI for the same Ragdoll cat with different postures and angles, including the front angle (upper) and the side angle (lower). d) The samples found by S‐XAI that satisfy the semantic condition proposed by humans. Left: dogs with a distinct nose. Right: cats without distinct legs.

In CNNs, the output of prediction is usually converted into the probability of each label by the softmax function, which will induce the problem of overconfidence since the softmax function exaggerates confidence.^[^ ^25^ ^]^ For example, for a photograph of a cat's back shown in **Figure** **5**a, a human may experience a challenge distinguishing it from a blanket, but the CNN recognizes it as a cat with a high probability of 97.7%. However, the semantic space extracted in this work can solve this problem since the semantic space and respective semantic probability are obtained from the information in the feature maps, without needing to go through the fully‐connected layers and softmax function, which means that the semantic probability is closer to the reality.

In this work, we extract six semantic spaces, including eyes, noses, and legs for cats and dogs, respectively. The semantic probabilities of these semantic spaces are illustrated by a radar map for each image. An explanation sentence is generated automatically by S‐XAI from the radar map utilizing different words to represent different levels of confidence (e.g., sure, probably, may, confusing, etc.). For example, a dog that is identified to be a dog with 100% probability by CNN is shown in Figure 5b. The S‐XAI can provide more information besides the classification probability given by the CNN. From the radar map, it is obvious that both the semantic probabilities of cats’ legs and dogs’ legs are high, which indicates that it is hard for the CNN to distinguish the legs of cats and dogs. In fact, if this dog's upper body is covered, humans cannot discern whether it is a dog or a cat by relying only on the legs. In addition, the semantic probabilities of dogs’ eyes and noses are dominant compared with cats’ eyes and noses. This information is reflected in the output of S‐XAI that “I am sure it is a dog mainly because it has vivid eyes and nose, which are dog's eyes and nose obviously. Although its legs are a little confusing.”

Considering that some big cats like Maine Coon cats are easy to be mistaken for a dog,^[^ ^28^ ^]^ we select three pictures of a big Ragdoll cat with different postures, including the front angle and the side angle (Figure 5c), and the back angle (Figure 5a). The output of CNN is similar and above 90% for the cats with all postures. However, radar maps obtained from the S‐XAI identify differences between these pictures. For the image in the front angle, the explanation of S‐XAI is “I am sure it is a cat mainly because it has vivid eyes and nose, which are cat's eyes and nose obviously. Meanwhile, it has vivid legs, which are something like cat's legs,” which shows high confidence. For the image in the side angle, the explanation of S‐XAI is “It is probably a cat mainly because it has eyes, which are perhaps cat's eyes. Although its legs are a little confusing.” For the image in the back angle, all semantic probabilities are inconspicuous, and the explanation of S‐XAI is “It may be a cat, but I am not sure.” These explanations made by S‐XAI are consistent with human cognition, in which from the front angle, it is universally identified to be a cat; whereas, its legs are confusing from the side angle, which may account for why big cats are frequently mistaken for a dog.

**Figure 6:**
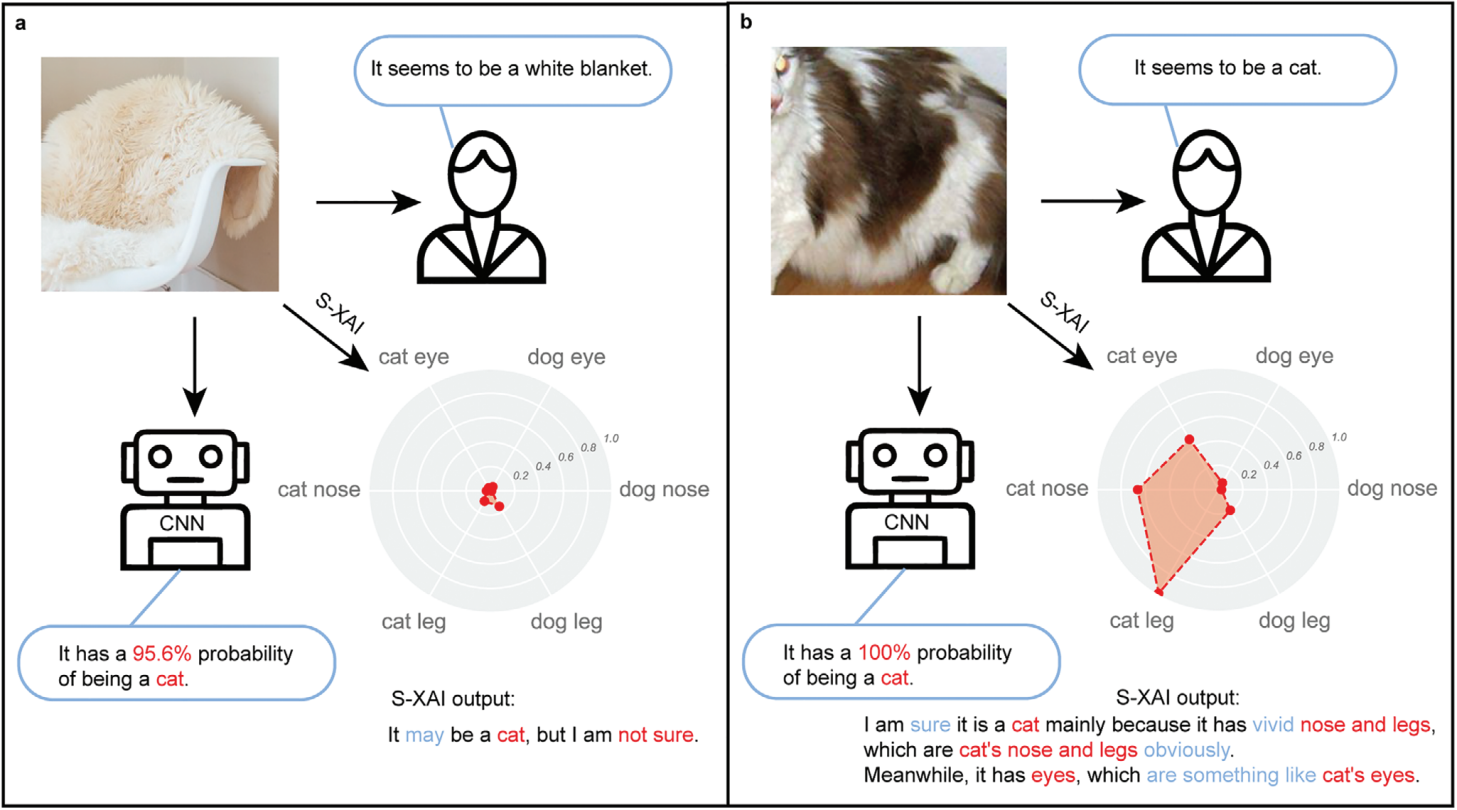
Assessments given by humans (right), CNN (bottom), and S‐XAI (bottom right) when identifying the picture of a white blanket (a) and a cat (b).

We also provide an example in which a white blanket is input into the CNN, which is shown in **Figure** **6**. While humans easily identify it as a white blanket instead of a cat, the CNN predicts that it has a 95.6% probability of being a cat, which is an incorrect assessment. The S‐XAI, however, discovers that all semantic probabilities of this image are low and outputs that “It may be a cat, but I am not sure,” which shows low confidence of the assessment. In contrast, when presented with a cat, S‐XAI can provide an explanation with high confidence.

The above experiments show that the explanation of S‐XAI provides more information to remedy the phenomenon of overconfidence and makes the process of prediction understandable for humans.

### 2.5 Application: Semantic Sample Searching

Since current research on semantic interpretation of neural networks is still in its infancy, finding target samples by semantics requires additional effort.^[^ ^26^ ^]^ However, once the semantic space is extracted, samples that satisfy semantic requirements through semantic probability can easily be found. For instance, if we want to find dogs with distinct noses in the dataset, we just need to set $P_{\mathrm{nose}}^{\mathrm{dog}}\left(z\right)>0.9$ and the discovered samples all have obvious noses, which is illustrated in Figure 5d. Similarly, images of cats without distinct legs can also be searched quickly by setting $P_{\mathrm{leg}}^{\mathrm{cat}}\left(z\right)<0.1$. The searching process is very fast because semantic probability in the semantic space can be calculated simultaneously during the prediction process of the neural network. This technique shows promising potential in the filtering of datasets and identifying “bad samples.”

### 2.6 Extendibility to Multiclassification Problems

**Figure 7:**
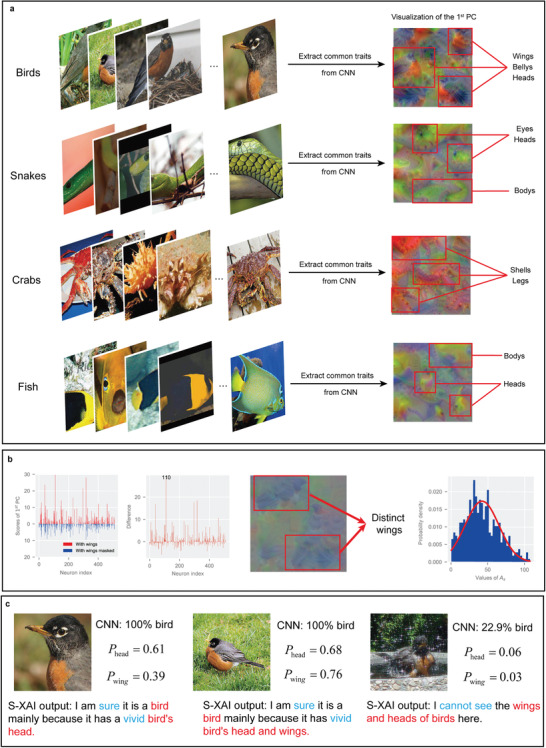
The extendibility of S‐XAI to multiclassification problems with the Mini‐ImageNet dataset. a) The extracted common traits of four example categories, including birds, snakes, crabs, and fish. b) The difference between the extracted common traits with and without masking birds’ wings, the extracted semantic space of birds’ wings, and the probability density distribution plot of the values of *A*_s_ for 400 samples of birds in the semantic space of birds’ wings. c) The S‐XAI output of semantic assessments for birds’ pictures from different angles.

In this work, a fundamental problem of binary classification, i.e., distinguishing cats and dogs, is taken as an example to show the ability of the proposed S‐XAI to provide semantic interpretations for the CNN. Considering that multiclassification problems are mainstream in practical applications, it is significant to examine the extendibility of the proposed S‐XAI to multiclassification problems with large datasets. Here, we adopt the Mini‐ImageNet dataset,^[^ ^29^ ^]^ which consists of 100 categories of objects. For each category, there are 600 samples, and thus the total number of pictures is 60 000. The VGG‐19 network is utilized here, and 50 000 samples are randomly selected to form the training dataset, while the other samples form the testing dataset. The classification accuracy of the trained CNN achieves 85%. The S‐XAI is adopted to interpret this network in multiclassification problems, and the results are displayed in **Figure** **7**.

The figure shows that the extracted common traits of different categories remain distinct and evident for multiclassification problems (Figure 7a). For example, the common traits of crabs from CNN present vivid shells and legs with the primary color paralleling crabs’ color. Meanwhile, the semantic spaces for each category can be extracted and visualized successfully in the same way (Figure 7b). Here, the semantic space of birds’ wings is taken as an example. It can be seen that the probability density distribution plot of *A*_s_ is also similar to a normal distribution. Finally, the extendibility of S‐XAI to give semantic assessments for CNN is examined. For the multiclassification problem, the rules to generate assessments need some slight adjustments by merely comparing the semantic probability of the target category, which is detailed in the Supporting Information S4. Figure 7c provides an example of giving semantic assessments for birds’ pictures from different angles, and it can be seen that the S‐XAI outputs are parallel with the truth. The experiments prove that the S‐XAI has good extendibility and can handle multiclassification problems.

## 3 Discussion

In this work, we proposed the framework of the semantic explainable AI (S‐XAI), which provides a global interpretation for CNNs by abstracting common traits from samples and extracting explicit understandable semantic spaces from CNNs. Statistical interpretation for the semantic space is further provided, and the concept of semantic probability is proposed for the first time. Experiments demonstrated that S‐XAI is effective in providing a semantic interpretation for the CNN, and has broad usage, including trustworthiness assessment and semantic sample searching.

In S‐XAI, the proposed RSC method plays a vital role, and can quickly extract highly hierarchical common traits from the feature maps of samples. Unlike conventional PCA that reduces feature dimensions, the proposed RSC reorganizes the original samples into a new reduced sample space, where each PC corresponds to a certain level of common traits. Then, the semantic concept is separated from the common traits through discovering the semantically sensitive neurons (SSNs) and their specific proportional relationship. On this basis, we visualize the semantic space for the first time. A set of orthogonal semantic concepts, including eyes, nose, and legs, is investigated in this work. It is found that the phenomenon of overlap of semantic spaces for CNN exists, which means that the semantic concepts in the CNN may be somewhat different from the definition of humans.

We also provide a statistical analysis for the extracted semantic space. The weighted average activation is defined in order to describe the activation of an image in semantic space, and it is discovered that the weighted average activation of sufficient natural samples is close to a normal distribution in the semantic space. Therefore, the concept of semantic probability is proposed for the first time, which can measure the likelihood of the occurrence of semantic concepts.

Our work goes a step further and investigates the application of our proposed S‐XAI by several experiments. The results show that the S‐XAI can provide trustworthiness assessment on the basis of semantic probabilities, which explores more information from the CNN, thus not only explaining the prediction made by the CNN, but also remedying the phenomenon of overconfidence. In addition, the S‐XAI is proven to be able to quickly search for samples that satisfy semantic conditions. The extracted semantic space also sheds light on the identification of adversarial examples, which may provide a potential way for adversarial example defense (Supporting Information S5). It is also proven that the proposed S‐XAI has good extendibility when adapting it to other structures of CNN and multiclassification tasks (more details are provided in the Supporting Information S4).

Overall, our study enables exploration of the representation of CNNs at the semantic level, and provides an efficient way to extract understandable semantic space. Compared with existing methods, the proposed S‐XAI can extract and visualize explicit semantic spaces without modifying the structure of CNN ( Supporting Information S8). The application of the S‐XAI framework to other deep learning areas is relatively straightforward, and is currently being explored. Further work also involves simplifying the process of extracting semantic space by adopting certain techniques, such as semantic segmentation^[^ ^30^, ^31^ ^]^ or annotation‐free techniques, for extracting object parts^[^ ^16^ ^]^ to mask semantic concepts automatically and making semantic concepts more explicit to obtain better semantic space. We believe that the S‐XAI will pave the way for understanding the “black box” model more deeply from the perspective of semantic space.

## 4 Experimental Section

### Superpixel Segmentation

Superpixel segmentation algorithms group pixels into perceptually meaningful atomic regions, which can be used to replace the rigid structure of the pixel grid.^[^ ^32^ ^]^ This greatly reduces the complexity of image processing tasks. Here, the simple linear iterative clustering (SLIC) technique^[^ ^32^ ^]^ based on k‐means clustering is adopted for superpixel segmentation, the detailed process of which is provided in the Supporting Information S7. Compared with most superpixel methods, SLIC has faster calculation speed, lower computational complexity, better stability, and is easy to implement in the python package *skimage*.

### Genetic Algorithm

The genetic algorithm is a well‐known optimization algorithm inspired from the biological evolution process.^[^ ^33^ ^]^ A typical genetic algorithm consists of crossover, mutation, fitness calculation, and evolution.^[^ ^34^ ^]^ In this work, a specific genetic algorithm is put forward to find the best combinations of superpixels based on the segmentation of superpixels that incurs the highest probability of the specified category from the CNN. For an image *z*, it was split into *N*_sp_ superpixels through the SLIC method and number each superpixel. A binary code of length *N*_sp_ is generated as the genome according to the superpixels, where 1 represents the existence of the superpixel and 0 represents nonexistence. For the initial generation, *N*_P_ genomes are randomly initialized. Then, the crossover process is conducted by randomly exchanging certain segments of two genomes, which leads to the occurrence of new genomes. Afterward, the genomes are mutated by randomly selecting genes and conducting bitwise negation. The fitness is then calculated to distinguish between good and bad genomes, which is defined as *P*_*i* = *c*_(*z*′) where *z*′ is the combination of superpixels translated from the genome, *c* is the target class, and *P_i = c_* is the output probability of the class *c* obtained from the CNN. Next, the genomes are sorted from large to small according to the calculated fitness. Finally, the first half of genomes remain, while others are replaced by new genomes in order to produce a new generation of parents. This process will cycle until the epoch achieves the maximum iteration that is set beforehand. In order to accelerate the convergence, the best genome is fixed until a better one replaces it in each generation. In this work, *N*_sp_ is 40, the population of genomes *N*_P_ is 50, the maximum iteration is 50, and the probability of mutation is 0.5.

### Dataset Settings

For binary classification problems, a well‐known dataset originated from the competition in Kaggle called Dogs v.s. Cats was used.^[^ ^35^ ^]^ This dataset includes 25 000 training images in which half are cats and half are dogs, and 12 500 test images. All of the images were rescaled into normalized 224 × 224 × 3 pixels to use the VGG‐19 network^[^ ^23^ ^]^ as a classifier. In the VGG‐19 network, a global average pooling (GAP) layer before the fully‐connected layer was added. The ImageNet‐pretrained weights as initial weights, as well as the Adam optimizer^[^ ^36^ ^]^ to train the neural network for three epochs through the mini‐batch technique with a batch size of 16 was used. The accuracy of the network on the test dataset is 97.5%. For multiclassification problems, the Mini‐ImageNet dataset,^[^ ^29^ ^]^ a subset of the ImageNet‐1K dataset, is used, which has 100 categories. For each category, there are 600 samples, and thus the total number of pictures is 60 000. Compared with the ImageNet‐1K dataset, which is bulky and contains a lot of unnecessary information for classification (e.g., anchor boxes for object detection), the Mini‐ImageNet dataset is more suitable for examining the extendibility of S‐XAI to multiclassification problems. The output neurons are 100, and other settings of CNN are the same as the binary classification situations. The classification accuracy is 85%.

### Row‐Centered Sample Compression

The row‐centered sample compression (RSC) method is based on the distinctive row‐centered PCA to compress sample dimensions in order to extract common traits of samples from CNN. The RSC method involves a dataset with observations on *p* numerical variables or features, for each of *n* individuals or observations, which forms an *n* × *p* data matrix *X*.^[^ ^27^ ^]^ In this work, *n* equals the number of samples *N*_s_, and *p* is the number of features which equals the number of channels in this work. For a conventional PCA that is usually utilized to reduce dimension, the columns of the data matrix are centered to calculate the covariance matrix, which facilitates dimension reduction after singular value decomposition (SVD). Therefore, the commonly‐used conventional PCA is also termed the column‐centered PCA. In this work, a rarely seen row‐centered PCA is adopted in the RSC method, which achieves good performance on extracting common traits. In the RSC method, the rows of the data matrix *X* are centered. The row‐centered data matrix $\hat{X}$ where ${\hat{X}}_{i,j}=X_{i,j}-{\bar{X}}_{i}$ is denoted, *i* is the index of rows, and *j* is the index of columns. The covariance matrix *S* is calculated as $S=\frac{1}{p-1}\hat{X}{\hat{X}}^{T}$. Then, the SVD is adopted to the covariance matrix *S*, and *S* = *U*∑*V^T^* is obtained where *U*, ∑, *V* ∈ *R* ^*n* × *n*^ . ∑ is a diagonal matrix, called the singular value matrix, where the diagonal elements (i.e., singular value) are arranged from largest to smallest. Since ∑ is a square matrix, the singular value equals the eigenvalue. It is worth noting that the rank of ∑ is *r* < min {*n*, *p*}, which means that there are only *r* nonzero singular values (or eigenvalues). Herein, the first *k* (*k* ≤ *r*) singular values are preserved and *U_k_* ∈ *R* ^*n* × *k*^ are obtained from *U* by retaining the first *k* columns. Finally, the reduced principal components (PCs) are obtained by *X_k_* = *X^T^U_k_* ∈ *R* ^*p* × *k*^, where each column of *X_k_* is a PC. For example, $X_{k}^{\left(\mathrm{col}=1\right)}$ is the 1st PC, $X_{k}^{\left(\mathrm{col}=i\right)}$ is the *i*th PC, and the elements of PC are called PC scores. The standard measure of quality of a given *i*th PC is the proportion of total variance (or the information ratio) that is calculated as $\pi_{i}=\lambda_{i}{\left(\sum_{i=1}^{p}\lambda_{i}\right)}^{-1}=\lambda_{i}{\left(\mathrm{tr}(S)\right)}^{-1}\times 100\%$, where tr(*S*) denotes the trace of the covariance matrix *S*, and *λ_i_* is the corresponding eigenvalue. Therefore, the quality of the retained *k*‐dimensional PCs can be expressed as a percentage of total variance accounted for: $\sum_{i=1}^{k}\pi_{i}$ . It is common practice to use some predefined percentage of total variance explained to decide how many PCs should be retained (70% of total variability is common), and the emphasis in PCA is almost always on the first few PCs.^[^ ^27^ ^]^

Feature Visualization

The feature visualization techniques aim to solve the following question: given an encoding feature of an image, to what extent is it possible to reconstruct the image itself?^[^ ^6^ ^]^ This question is closely related to the explanation of the network since the visualized feature map can tell us what kinds of features are learned by the network. The core issue of feature visualization is solving the minimization problem: $z^{*}=\underset{z\in R^{C\times H\times W}}{\arg\;\min}\left(L(\Phi \left(z\right),\Phi_{0})+\lambda R\left(z\right)\right)$, where *L* is the loss function which is usually mean squared error (MSE), Φ_0_ is the target encoding feature, and Φ(*z*) is the corresponding feature of optimized image *z* with the size of *C*×*H*×*W* obtained from the network, where *C* is the number of channels, *H* and *W* are the height and width of the feature map, respectively, and *R* is the regularization term capturing a natural image prior. The focal point of the minimization is the selection of *R*, and thus leads to different techniques of feature visualization, including L_2_‐regularization,^[^ ^6^ ^]^ total variation (TV) regularization,^[^ ^6^ ^]^ and deep image prior regularization.^[^ ^10^ ^]^ The existence of *R* prevents the minimization process from converging to the images of high frequency that humans cannot discern. In this work, MSE loss for *L* and total variation (TV) regularization for *R* are utilized. Therefore, the minimization problem is converted into the following

(5) $$z^{*}=\underset{z\in R^{C\times H\times W}}{\arg\min}\left({\left\|\Phi \left(z\right)-\Phi_{0}\right\|}^{2}+\lambda R\left(z\right)\right)$$

(6) $$R\left(z\right)=\sum_{k}{\sum_{i,j}\left({\left(z_{k,i,j+1}-z_{k,i,j}\right)}^{2}+{\left(z_{k,i+1,j}-z_{k,i,j}\right)}^{2}\right)}^{\frac{\beta }{2}}$$

where *λ* and *β* are employed to control the magnitude of regularization. The gradient descent technique is used to solve this minimization problem as follows

(7) $$z^{n+1}=z^{n}-\delta \cdot \frac{d\left({\left\|\Phi \left(z^{n}\right)-\Phi_{0}\right\|}^{2}+\lambda R\left(z^{n}\right)\right)}{dz^{n}}$$

where *z^n^* is the image after the *n*th iteration; *δ* is the learning rate; and the initial image is defined as a blank figure with all elements equal to zero after standardization. The optimization process will continue until the epoch reaches the maximum iteration. In this work, *λ*, *β*, and *δ* are set to be 2, 2, and 0.05, respectively. The learning rate *δ* is multiplied by 0.5 for each 1000 epochs. The maximum iteration is 4000.

In previous works,^[^ ^6^, ^10^, ^11^ ^]^ feature visualization techniques are usually employed to visualize the feature maps in the network, which means that they can only analyze what is learned by the network for each image. Although they enable understanding the feature learned by the network in each layer, they are also constrained to be a local explanation. In this work, on the visualization of each single image is not focused, but rather visualization of the principal components obtained by the row‐centered sample compression that contains common traits, thus providing a global interpretation. Therefore, the Φ_0_ is each PC and the optimized image *z* is the visualization of the PC, which contains common traits in this work.

### Extraction of Common Traits

With the assistance of the RSC method, it is possible to extract common traits from the samples of the same class. Common traits are extracted for cats as an example (see Figure 1a). *N*_s_ cat samples from the dataset are first randomly selected, and the best combination of superpixels for each sample via the SLIC technique is discovered. Subsequently, the samples are fed into the network, and the feature maps of the last layer before the fully‐connected classifier are extracted. The size of the feature map for each sample is a *C*×*H*×*W* matrix. Specifically, the feature map is degenerated to a vector with the length of *C* in this work since the last layer is the global average pooling (GAP) layer. Consequently, the feature maps for the selected *N*_s_ cat samples form a matrix with the size of *N*_s_×*C*. In order to extract common traits, the RSC method is adopted. Considering that the number of channels *C* equals the number of features *p* in this work, the matrix of feature maps is seen as the data matrix with the size of *N*_s_×*p*. In the RSC, we preserve *k* PCs that make the percentage of total variance achieve 85% of the total variability and obtain a *k* × *p* matrix after PCA. In this work, the first few PCs are primarily focused on, since the information ratios of others are negligible. This RSC method can also be utilized for other layers or networks easily (Supporting Information S2). After the RSC method, the feature visualization technique is adopted to visualize each PC to exhibit the common traits in a human‐understandable way (see the right of Figure 1a).

### Masking the Semantic Concept

In order to explore the semantic space, it is necessary to mask the semantic concept, such as eyes, nose, and legs, in the image (see the left of Figure 1b). In this work, it is completed manually since the semantic concept is defined by humans. To improve the efficiency of the masking process, a program is designed to assist manual processing. For an image *z*, it is firstly split into *N*_sp_ superpixels (*N*_sp_ = 20 here). Then, the superpixels that contain the target semantic concept are selected manually, and are filled with the color of a nearby superpixel that is also selected manually. This process will mask the semantic concept, thus obtaining the same image without the target semantic concept. In this work, we have shown that 100 images with and without the target semantic concept are sufficient for extracting the semantic space, which means that manual processing is acceptable here. However, when faced with extraction of a large‐scale semantic space with numerous semantic concepts and categories, manual processing is inefficient and some other techniques, such as semantic segmentation^[^ ^30^, ^31^ ^]^ or annotation‐free techniques, for extracting object parts^[^ ^16^ ^]^ may be viable approaches to improve this process.

### Extraction and Visualization of Semantic Space

In this work, for the first time, a simple yet effective method to extract and visualize semantic space in an understandable manner is presented. Here, the method in detail will be introduced. The method is constructed based on the extracted common traits of the images with and without the target semantic concept (see Figure 1b). First, *N*_s_ samples with the explicit target semantic concept are selected, and then the target semantic concept is masked to generate samples without the target semantic concept (*N*_s_ = 100 in this work). For masked and unmasked samples, the common traits are extracted in the same way as that mentioned above, respectively. It is worth noting that it is not needed to search the best combinations of superpixels here since the semantic concept is focused on. Considering that the information ratio of the 1st PC is dominant compared with that of the others, which means that it contains more information of common traits, the 1st PC here is only analyzed. The difference between both common traits is calculated as *s*_unmask_‐*s*_mask_, where *s*_mask_ and *s*_unmask_ are the scores of the 1st PC for the samples with masked and unmasked semantic concept, respectively. From the comparison, it is found that several neurons are particularly sensitive to the existence of the semantic concept, which are termed as semantically sensitive neurons (SSNs) in this work. *N*_SSN_ semantically sensitive neurons with the largest absolute difference are chosen. It is worth mentioning that the absolute difference of these SSNs constitutes a specific proportional relationship. The extracted semantic space is made up of both the SSNs and the specific proportional relationship. In this work, *N*_SSN_ is set to be 5. In order to visualize the extracted semantic space, a target Φ_0_ is designed for visualization, where its size is the same as the feature map in the last layer before fully‐connected layers, and all elements are zeros except the discovered SSNs. For the discovered SSNs in Φ_0_, the values obey the specific proportional relationship with the maximum value enlarged (to 30 in this work). Through the visualization of Φ_0_, the semantic space is exhibited in a human‐understandable way (see the right of Figure 1b).

### Trustworthiness Assessment

The proposed S‐XAI is able to provide more information and make the trustworthiness assessment based on the extracted semantic space. The trustworthiness assessment includes two components: a radar map of semantic probabilities; and a trustworthiness assessment. The radar map depicts the semantic probabilities in different semantic spaces. Through the radar map, a substantial amount of information can be observed to produce the trustworthiness assessment. Here, for an image *z*, the semantic space of the predicted class given by the CNN is first focused on, in which the maximum semantic probability is denoted as *P*_max_(*z*) and the respective semantic concept is denoted as *S*_max_. Then, for each semantic concept, the class that has the maximum semantic probability (except the predicted class) is discovered, and the difference of the semantic probabilities between the predicted class and the discovered class are defined as Δ*P*(*z*). Among Δ*P*(*z*) for all semantic spaces, the maximum is termed as Δ_max_ *P*(*z*). The standard of a trustworthiness assessment is determined by *P*_max_(*z*), Δ*P*(*z*), and Δ_max_ *P*(*z*), which identify the confidence by using different words (e.g., sure, probably, may, confusing, etc.). The rules for generating the explanation by S‐XAI is provided in **Table** **1**. The rules to generate S‐XAI output are not restricted and are determined empirically in this work. An empirical criterion can work well in this work because S‐XAI essentially provides a qualitative explanation from a semantic perspective. It is worth noting that the explanation given by S‐XAI is generated automatically without any additional information or manual processing. Once the semantic spaces have been extracted and the distribution of the weighted average activation has been fixed, the proposed S‐XAI can make the trustworthiness assessment and provide a corresponding explanation as soon as the network makes the prediction.

**Table 1:** Rules for generating the explanation by S‐XAI

	Δ_max_ *P*(*z*)<0.2	0.2<Δ_max_ *P*(*z*)<0.35	0.35<Δ_max_ *P*(*z*)<0.5	Δ_max_ *P*(*z*)>0.5
Assessment	It might be a dog/cat, but I am not sure.	It is probably a dog/cat mainly because		I am sure it is a dog/cat mainly because
Explanation				
	Δ*P*(*z*)<0.2	0.2<Δ*P*(*z*)<0.35	0.35<Δ*P*(*z*)<0.5	Δ*P*(*z*)>0.5
*P*_max_(*z*)>0.5	Position: vivid, Semanteme: confusing	Position: vivid, Semanteme: perhaps	Position: vivid, Semanteme: something like	Position: vivid, Semanteme: obviously
0.2<*P*_max_(*z*)<0.5	None	Position: be, Semanteme: perhaps	Position: be, Semanteme: something like	Position: be, Semanteme: obviously

## Conflict of Interest

The authors declare no conflict of interest.

## Author Contributions

H.X., Y.C., and D.Z. conceived the project, designed, and performed research, and wrote the paper; H.X. implemented workflow, created code, visualized results, and analyzed and curated data.

## Supporting information

Supporting Information

## Data Availability

The code to reproduce the figures and results of this article can be found at https://github.com/woshixuhao/semantic‐explainable‐AI. The pre‐trained models and some results are available at Google Drive Share: https://drive.google.com/file/d/1sXPUR3dwBE1HjykqO4ftwpAIHCqErY16/view?usp=sharing https://drive.google.com/file/d/1Eythcx_wj‐_eZ9JIaQtKwHyITzEKwtQ5/view?usp=sharing.
