# Supplementary material for: Semantic Interpretation for Convolutional Neural Networks: What Makes a Cat a Cat?
Source: Adv Sci (Weinh). 2022 Oct 10;9(35):2204723. doi: 10.1002/advs.202204723 (PMC9762288; doi:10.1002/advs.202204723)
Supplement: Supplementary file 1 — Supporting Information [file ADVS-9-2204723-s001.pdf]

ADVANCED SCIENCE
Open Access

Supporting Information

for *Adv. Sci.*, DOI 10.1002/advs.202204723

Semantic Interpretation for Convolutional Neural Networks: What Makes a Cat a Cat?

Hao Xu, Yuntian Chen and Dongxiao Zhang**

Supplementary Information for

Semantic interpretation for convolutional neural networks: What makes a cat a cat?

Hao Xu[a], Yuntian Chen[b, *], and Dongxiao Zhang[c,d,*]

[a] *BIC-ESAT, ERE, and SKLTCS, College of Engineering, Peking University, Beijing 100871, P. R. China*

[b] *Eastern Institute for Advanced Study, Yongriver Institute of Technology, Ningbo 315200, Zhenjiang, P. R. China*

[c] *National Center for Applied Mathematics Shenzhen (NCAMS), Southern University of Science and Technology, Shenzhen 518055, Guangdong, P. R. China*

[d] *Department of Mathematics and Theories, Peng Cheng Laboratory, Shenzhen 518000, Guangdong, P. R. China*

[*] Corresponding author.

The PDF file includes:

S.1: Supplementary information for the extraction of common traits.

S.2: Extension of the proposed row-centered sample compression to general feature maps.

S.3: Supplementary information for the statistical explanation of semantic space.

S.4: Extendibility of the S-XAI to other structures of CNN and multi-classification tasks.

S.5: Adversarial example identification.

S.6: Supplementary discussions.

S.7: Details of superpixel segmentation.

S.8: Comparison with existing methods.

Supplementary Fig. 1. Comparison between the common traits extracted from samples with and without experiencing the genetic algorithm.

Supplementary Fig. 2. Visualization of the last PC in the row-centered sample compression.

Supplementary Fig. 3. Scores of the 1st PC with different N_s utilizing different random seeds to select different samples.

Supplementary Fig. 4. The information ratio of the 1st PC for each layer in the network.

Supplementary Fig. 5. Probability density distribution plots and q-q plots for different semantic spaces.

Supplementary Fig. 6. The results of S-XAI for AlexNet.

Supplementary Fig. 7. Assessments given by humans, CNN, and S-XAI when identifying the true sample and the adversarial example.

Supplementary Fig. 8. Comparison with existing CNN interpretation methods regarding feature visualization.

Supplementary Table 1. The symbols used in this work and their meanings.

Supplementary Table 2. The success rate of attack by PGD and the success rate of defense by S-XAI with different attack strengths.

Supplementary Table 1. The symbols used in this work and their meanings.

Symbol	Meaning
N_s	The number of selected samples
N_SSN	The number of semantically sensitive neurons
$A_s(z)$	The weighted average activation of image z
$\mathrm{P}_s(z)$	The semantic probability of image z in semantic space s
N_e	The number of repeated experiments
p	The number of features
C	The number of channels
H, W	The height and width of the feature map, respectively
N_sp	The number of superpixels
N_P	The initial population of genomes
$P_{i=c}(z)$	The output probability of the class c obtained from CNN
e	Spread from the average

S.1: **Supplementary information for the extraction of common traits**

In this work, a specific genetic algorithm is utilized to obtain the optimal combinations of superpixels for each sample. Here, an experiment is conducted to compare the common traits extracted from samples with and without experiencing the genetic algorithm, the results of which are displayed in Supplementary Fig. 1. From the figure, it is obvious that the common traits extracted from the best combinations of superpixels discovered by the genetic algorithm present more explicit semantic concepts compared with those without experiencing the genetic algorithm, which proves that the genetic algorithm assists to reduce interference and makes the extracted common traits more representative.

From the visualization of the 1st, 2nd, and 3rd PCs after the row-centered sample compression, it is discovered that different PCs present traits at different levels. Considering that the first several PCs contain a large number of common traits, it is interesting to visualize the last PC, which is shown in Supplementary Fig. 2. Here, we retain 299 PCs from 300 samples and the last PC is the 299th PC. From the figure, high-heeled shoes and a medicine bottle that constitute the background of the main images emerge in the visualization, which implies that the information ratio is closely related to the concentration of the common features.

Here, we also provide the scores of the 1st PC with different N_s utilizing different random seeds to select different samples, and the results are shown in Supplementary Fig. 3. From the figure, it can be seen that the 1st PC is more stable when the N_s is larger. Furthermore, it is discovered that the scores of the 1st PC exhibit a trend of proportional expansion and maintain a constant proportional relationship between the scores when N_s increases. This implies that the constant proportional relationship determines the content of the common traits, while the magnitude of the scores determines the number of common traits presented by the visualization.

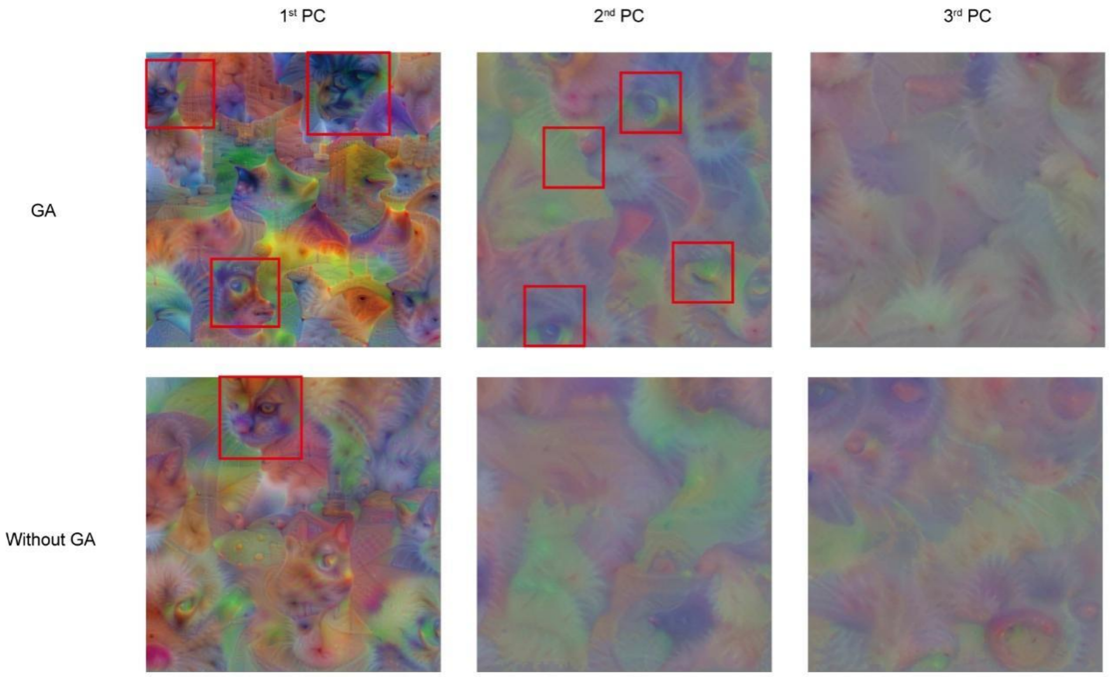

Supplementary Fig. 1. Comparison between the common traits, including the 1st PC, 2nd PC, and 3rd PC extracted from samples with and without experiencing the genetic algorithm. The red frame refers to the semantic concepts that can be recognized explicitly.

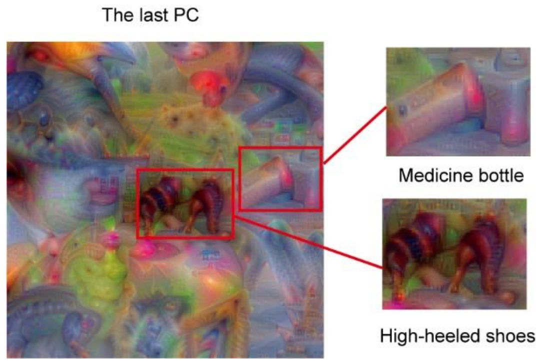

Supplementary Fig. 2. Visualization of the last PC (left) in the row-centered sample compression and partial enlarged pictures (right).

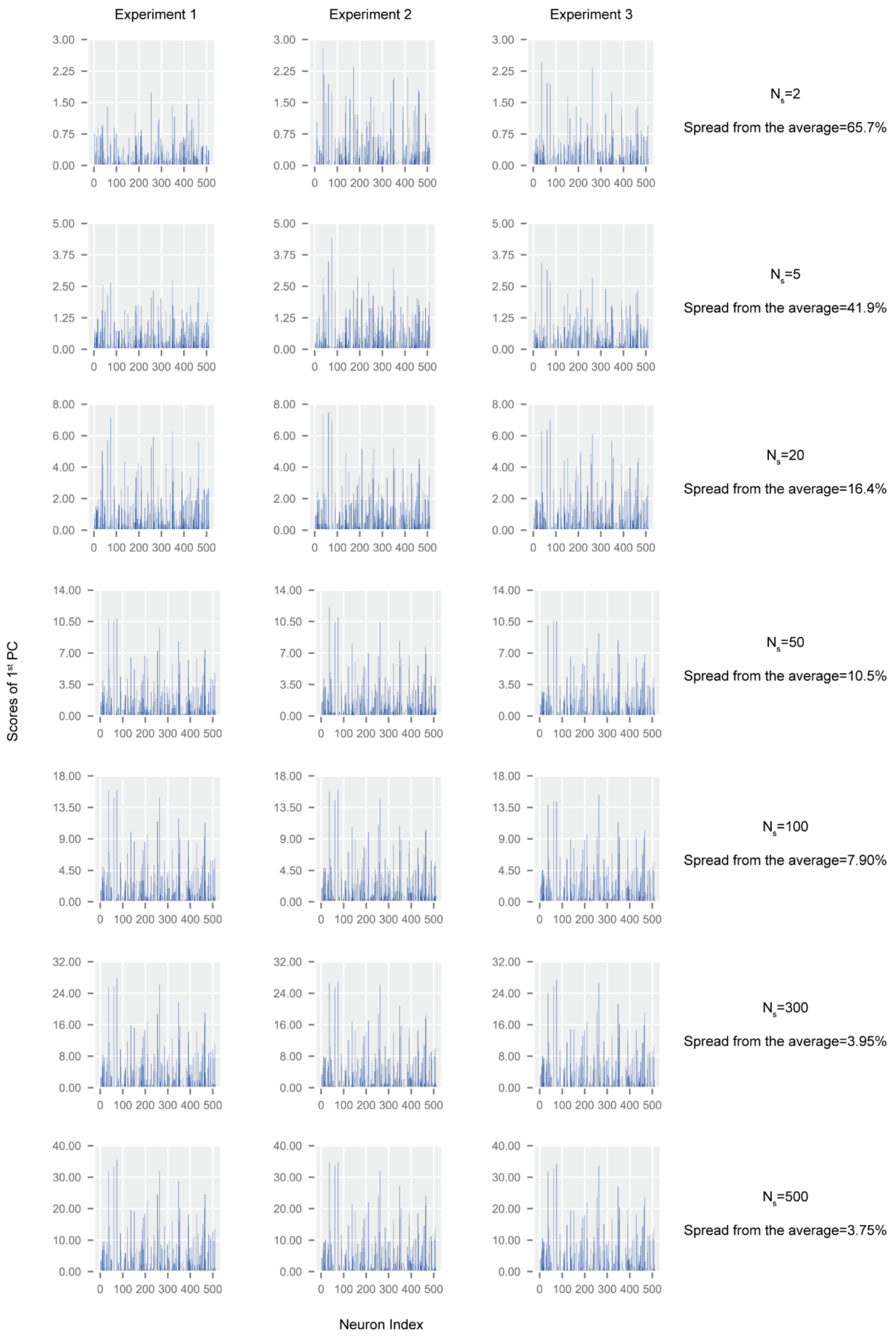

Supplementary Fig. 3. Scores of the 1st PC with different N_s utilizing different random seeds to select different samples.

S.2: Extension of the proposed row-centered sample compression to general feature maps

In this work, a row-centered sample compression (RSC) method is utilized to extract and visualize common traits of samples from CNN. In this section, the extension of the proposed RSC method to general feature maps is investigated. The size of the feature map for each sample is a $C\times H\times W$ matrix, where C is the number of channels, and H and W are the height and width of the feature map, respectively. In this work, the feature map is degenerated to a vector with the length of C since the last layer is the global average pooling (GAP). In fact, the RSC method can be extended to common feature maps generally. For N_s feature maps extracted from N_s samples, the data matrix is $N_s\times C\times H\times W$. The RSC for this data matrix can be conducted in the $N_s\times C$ submatrix for each point in the $H\times W$ submatrix. For the $H\times W$ times of the RSC, we uniformly retain k principal components and obtain the reduced submatrix with the size of $k\times C$. Finally, we make the $k\times C$ and $H\times W$ submatrices concrete to obtain the ultimate $k\times C\times H\times W$ matrix after the RSC.

Here, we conduct the RSC on the feature maps of each layer in the network to further explore the differences between different layers, and the information ratio of the 1st PC for each layer is illustrated in Supplementary Fig. 4. It is found that the information ratio of the 1st PC exhibits a trend of first decreasing and then increasing with the deepening of layers. Particularly, the last global average pooling (GAP) layer greatly promotes the information ratio of the 1st PC, which proves that it can realize dimension reduction, preserve spatial information extracted by the previous convolutional layers and pooling layers, and thus concentrate the common traits. Meanwhile, considering that the information ratio of the 1st PC is closely related to the extraction of common traits, the convolutional layer and max pooling layer seem to contribute to concentrating the common traits, which may explain the powerful generalization ability of CNNs. The trend of information ratio of the 1st PC also provides solid evidence that the shallow layers of the CNN acquire simple texture features that have more common traits, the middle layers acquire local features that have fewer common traits, and the deep layers acquire overall category information, i.e., semantic information, that has more common traits.

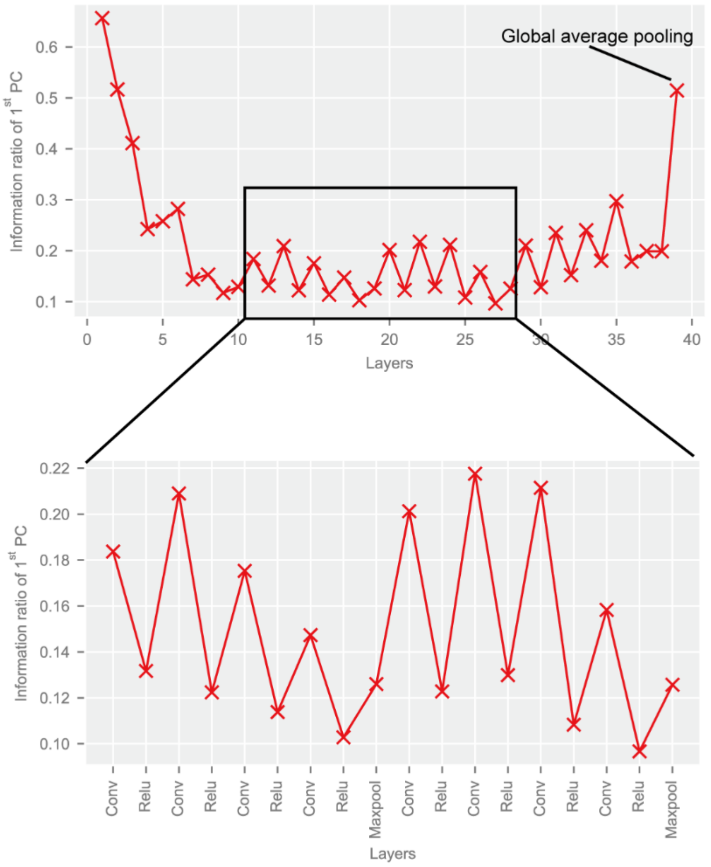

Supplementary Fig. 4. The information ratio of the 1st PC for each layer in the network (upper) and partial enlarged pictures (lower). In the upper part, the label of the x-axis is the layer index, and the last layer is the global average pooling (GAP) layer. In the lower part, the label of the x-axis is the class of the layer.

S.3: Supplementary information for the statistical explanation of semantic space

In this work, the distribution is close to part of the normal distribution in the semantic space of cats' eyes. Here, we provide the probability density distribution plots of the values of the weighted average activation A_s for 3,000 samples of cats and dogs in different semantic spaces, including eyes, nose and legs, which are displayed in Supplementary Fig. 5. The respective quantile-quantile (q-q) plot, which is a graphical technique for determining if the given distribution is consistent with a normal distribution, is used. From the q-q plots, it is discovered that the R^2 are all above 0.97, which shows a strong correlation between the distribution and normal distribution in all semantic spaces. It is also worth noting that the distributions are consistent with the fitted normal distribution in the latter half, which proves that the distributions of semantic spaces are close to part of the normal distribution. Moreover, it is interesting to find that the distribution of the weighted average activation is actually close to part of the normal distribution and exhibits a slight difference. This is because the selected 3,000 samples cannot fully represent the concept of "cat" or "dog", which means that the dataset itself is imperfect. It is also worth mentioning that the semantic concepts here can be defined arbitrarily in a way that humans can understand, which enhances the interpretability of the CNN.

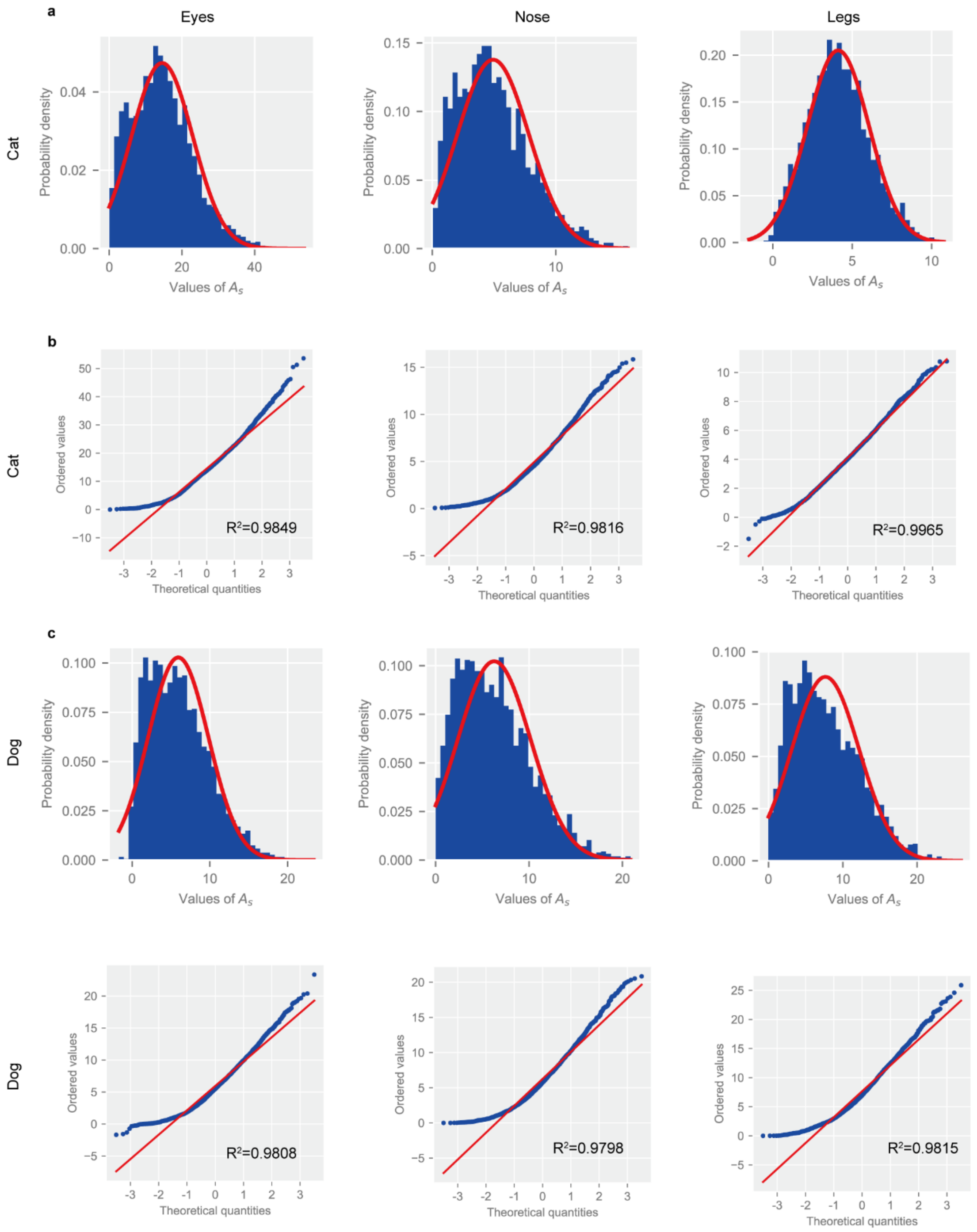

Supplementary Fig. 5. Probability density distribution plots of the values of the weighted average activation A_s for 3,000 samples of cats (a) and dogs (c) in different semantic spaces, including eyes (left), nose (middle) and legs (right), where the red curves are the fitted normal distributions and the quantile-quantile (q-q) plots of the distribution for cats (b) and dogs (d), including eyes (left), nose (middle) and legs (right), and where the red lines are the quantile lines of the fitted normal distributions.

S.4: Extendibility of the S-XAI to other structures of CNN and multi-classification tasks

In this section, we will provide more information about the extendibility of the S-XAI to other structures of CNN and multi-classification tasks.

In this work, the VGG-19 network with a global average pooling (GAP) layer is employed mainly because the visualization of layers in the VGG-19 network has been proven to be effective and recognizable[1,2]. Here, we investigate the extendibility of our proposed S-XAI to other structures of CNN. We adopt the S-XAI to AlexNet with a GAP layer, and the results are shown in Supplementary Fig. 6. From the figure, it can be seen that S-XAI can also extract the semantic space in AlexNet, but accuracy is affected. First, the visualization of common traits extracted from AlexNet is so abstract that the semantic information is not explicit enough to be recognized like that in the VGG-19 network. In fact, in previous work, the visualization of AlexNet proves the existence of the phenomenon of distortion and abstraction[2]. However, the distorted silhouette still reveals some common traits that can differentiate cats and dogs. From the probability density distribution plot of the values of the weighted average activation and the q-q plot, it is discovered that although the distribution deviates more from the normal distribution compared with the VGG-19 network, the semantic probability can still represent the probability of the semantic concept well, which means that the semantic space is extracted successfully by S-XAI from AlexNet. In addition, it is found that the semantically sensitive neurons are similar for the cats' legs and dogs' legs in the semantic space, which makes it difficult to differentiate the cats' legs and dogs' legs in the semantic space. This suggests that AlexNet may not be highly sensitive to the legs of dogs and cats, which indicates that the ways of extracting semantic spaces may be different in different constructions of CNNs.

For multi-classification tasks, the rules for generating semantic assessments need to be adjusted slightly. Here, we focus on explaining how CNN recognizes specific categories from the aspect of semantics. For each category, it is supposed that N_{sc} semantic concepts can be extracted. For each semantic concept, the corresponding semantic probability P_i can be calculated. In this work, if the maximum of P_i, P_{max}, is larger than 0.5, the assessment is 'I am sure it is…'. If $0.2<P_{max}<0.5$, the assessment is 'It is probably…'. If $P_{max}<0.2$, the assessment is 'I cannot see…'. For each semantic concept, if the semantic probability $P_i>0.5$, the description is 'vivid'. If $0.35<P_i<0.5$, the description is 'something like'. If $0.2<P_i<0.35$, the description is 'perhaps'. If the semantic probability $P_i<0.2$, no description is displayed. It is worth noting that if the difference between the semantic concept with the maximum P and the others is larger than 0.2, the S-XAI only outputs the central semantic concept with the maximum P.

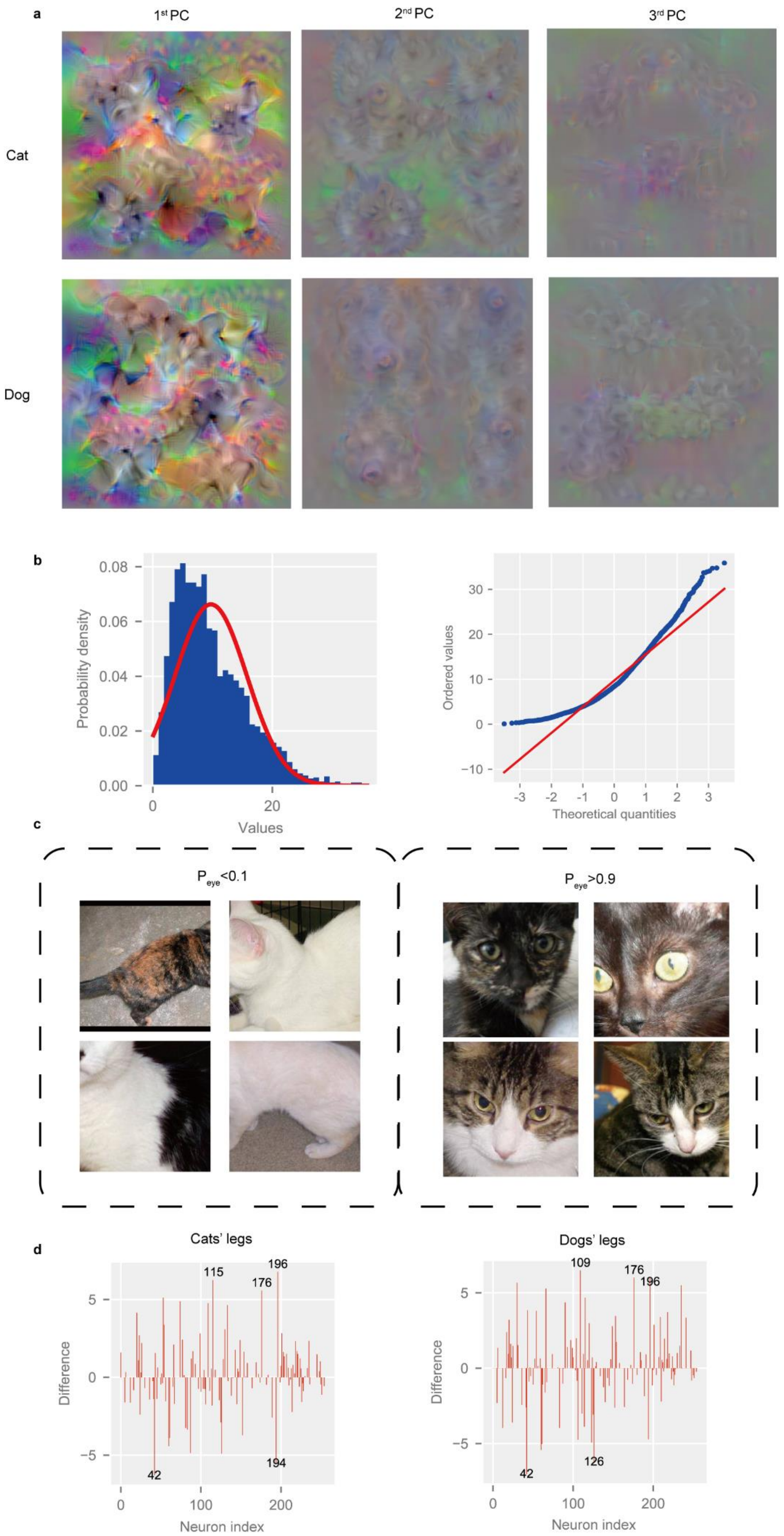
a
1st PC
2nd PC
3rd PC
Cat
Dog
b
Probability density
Values
Ordered values
Theoretical quantities
c
P eye<0.1
P eye>0.9
d
Cats' legs
Dogs' legs
Difference
Neuron index
115
196
176
42
194
109
126

Supplementary Fig. 6. The results of S-XAI for AlexNet. **a,** Visualization of the 1st, 2nd, and 3rd PCs for cats and dogs with N_s=300, respectively. **b,** Probability density distribution plot of the values of the weighted average activation A_s for 3,000 samples of cats in the semantic spaces of cats' eyes (left) where the red curve is the fitted normal distribution curve, and the quantile-quantile (q-q) plot of the distribution (right) where the red line is the quantile line of the fitted normal distribution. **c,** Samples located at the left (P_{eye}<0.1) and right (P_{eye}>0.9) ends of the distribution, respectively. **d,** The difference for the cats' legs (left) and dogs' legs (right), where the notations are the first five semantically sensitive neurons.

S.5: Adversarial example identification

Recently, many studies have been performed on adversarial samples, including L-BFGS[3], FCSM[4], PGD[5], etc., the purpose of which is to impose noise that cannot be discerned by human eyes on the samples to reduce the confidence of the neural network and induce incorrect assessments, which poses a challenge for the neural network to guarantee its safety[6]. Methods of defense against adversarial examples have also been extensively studied[7–10], the most notable of which is adversarial training that improves the robustness of DNNs against adversarial attacks by retraining the model on adversarial examples[4]. However, it is costly to retrain the neural network.

In this work, we attempt to understand the adversarial example and identify it from the perspective of semantic space. PGD is chosen to attack the CNN as an example. By comparing the probability density distribution of natural samples and adversarial samples in the semantic space, it is found that the location of adversarial samples is very close to the right end of the fitted normal distribution of natural samples, which means that the semantic probability of adversarial samples is very close to 1 or even greater than 1. The radar maps of the true sample and adversarial sample are provided in Supplementary Fig. 7, which reveals that the semantic probabilities of the adversarial examples are unusually large compared with the true samples. This is because in order to achieve a high attack success rate, the adversarial samples often contain excessive information about the incorrect label, which means that its activation in semantic space is much higher than that in natural samples. Therefore, the semantic space can identify adversarial examples to a certain extent, the results of which are shown in Supplementary Table 2. The criterion for identifying the adversarial example is simple, in which one of the semantic probabilities is higher than 0.99 or more than one of the semantic probabilities is higher than 0.9. From the results, the accuracy of identification of adversarial examples shows that the stronger is the adversarial attack, the higher is the success rate of identification by S-XAI; whereas, the weaker is the adversarial attack, the lower is the success rate of identification by S-XAI. This demonstrates that the identification of adversarial examples via the semantic space limits the strength of adversarial attacks, so that the attacker has to incur a greater cost to find suitable parameters to control the strength of adversarial attacks. Considering that the attack methods of adversarial samples emerge in an endless stream, the proposed method is not invulnerable. However, because of its low defense cost, it is highly suitable to be integrated into defense methods to improve the success rate of defense. Overall, the semantic space sheds light on the defense of the adversarial example, and better defense techniques may be inspired from semantic space in the future.

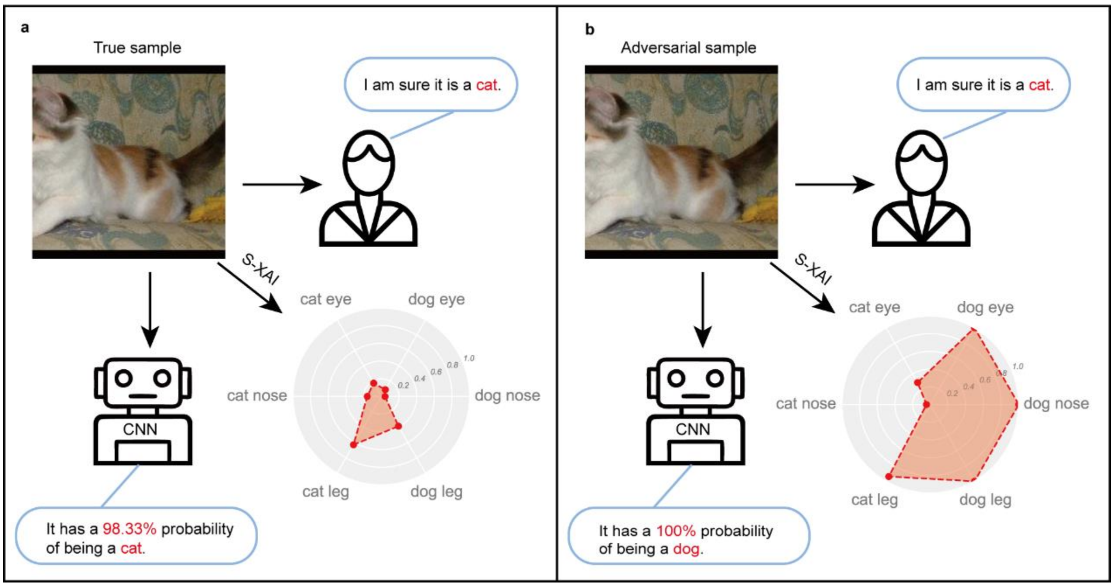

Supplementary Fig. 7. Assessments given by humans (right), CNN (bottom), and S-XAI (bottom right) when identifying the true sample (a) and the adversarial example (b).

Supplementary Table 2. The success rate of attack by PGD and the success rate of defense by S-XAI with different attack strengths.

**Parameters**	**ε=0.05**	**ε=0.01**	**ε=0.005**
Success rate of attack	100	100	84
Success rate of defense	100	38	0

S.6: Supplementary discussions

Although semantic spaces are successfully extracted in this work, certain shortcomings remain in the current research. First, the extraction of semantic space requires masking the semantic concepts in samples, which is completed manually. Although we have experimentally proven that only 100 masked images are sufficient to extract the semantic space well, manual annotation is still a major limitation, especially when faced with large-scale semantic space extraction with numerous semantic concepts and categories. Some other techniques, such as semantic segmentation or annotation-free techniques, for extracting object parts may assist to solve this problem. Second, numerous semantic concepts exist that are difficult to be masked in the image. For example, in this work, we use a set of typical semantic concepts to explain the CNN, including eyes, nose, and legs. However, certain infrequent semantic concepts, such as paws, beards or tails, are difficult to include.

S.7: Details of superpixel segmentation

The detailed process of superpixel segmentation is provided here. First, the image is converted into CIELAB color space $[l, a, b]^T$. Then, k_s seed points (or cluster centers) are randomly initialized by sprinkling k_s points on the image on average, which evenly fills the entire image. For the 3×3 area centered on each seed point, the gradient value of each pixel is calculated, and the point with the smallest value is selected as the new seed point, which aims to prevent the seed

point from falling on the outline boundary. Afterwards, distance metrics for all pixels in the 2S*2S square area around the seed point are calculated, where S= $\sqrt{\frac{N_{sp}}{k_s}}$ and N_{sp} is the number of image pixels. The distance metric D between the pixels i and j is as follows:

$$
\begin{aligned}
D &= \sqrt{\left(\frac{d_c}{N_c}\right)^2 + \left(\frac{d_s}{N_s}\right)^2} \\
d_c &= \sqrt{(l_j - l_i)^2 + (a_j - a_i)^2 + (b_j - b_i)^2} . \\
d_s &= \sqrt{(x_j - x_i)^2 + (y_j - y_i)^2}
\end{aligned}
$$

where d_c is the color metric; d_s is the spatial metric; and $[x, y]^T$ is the pixel position. Since each pixel on the image may have several distance metrics calculated by different seed points, the seed point corresponding to the smallest distance metric is selected as its cluster center. This process will continue until the residual reaches the threshold set beforehand.

S.8: Comparison with existing methods

As demonstrated in the main text, there are three mainstream ways to interpret CNN, including feature visualization, network diagnosis, and structure modification. In this section, we will compare our proposed S-XAI with these methods.

Firstly, feature visualization is the most straightforward way to see what the CNN learns when classifying samples. Here, we compare the S-XAI with two mainstream ways, including gradient descent with total variance regularization (GDTV) and deep image prior[1,2], and the results are provided in Supplementary Fig. 8. These methods are devoted to visualizing the feature map of individual samples to show the feature ‘seen’ by CNN. From the figure, it is evident that both methods can present vague features, which can interpret CNN to some extent. However, the semantic concepts are unclear, and the visualized features are individual traits. In comparison, the common traits extracted by S-XAI from CNN are distinct and contain abundant semantic information. Secondly, for network diagnosis methods, the semantic information is recessive and can only be reflected by diagnosing a pre-trained CNN (e.g., Grad-CAM). In contrast, the proposed S-XAI can extract and visualize explicit semantic space, which is straightforward and easy to understand. Finally, compared with structure modification methods that adjust the structure of CNN for better interpretability, our proposed S-XAI does not need to adjust the CNN structure, which is superior regarding practical applications.

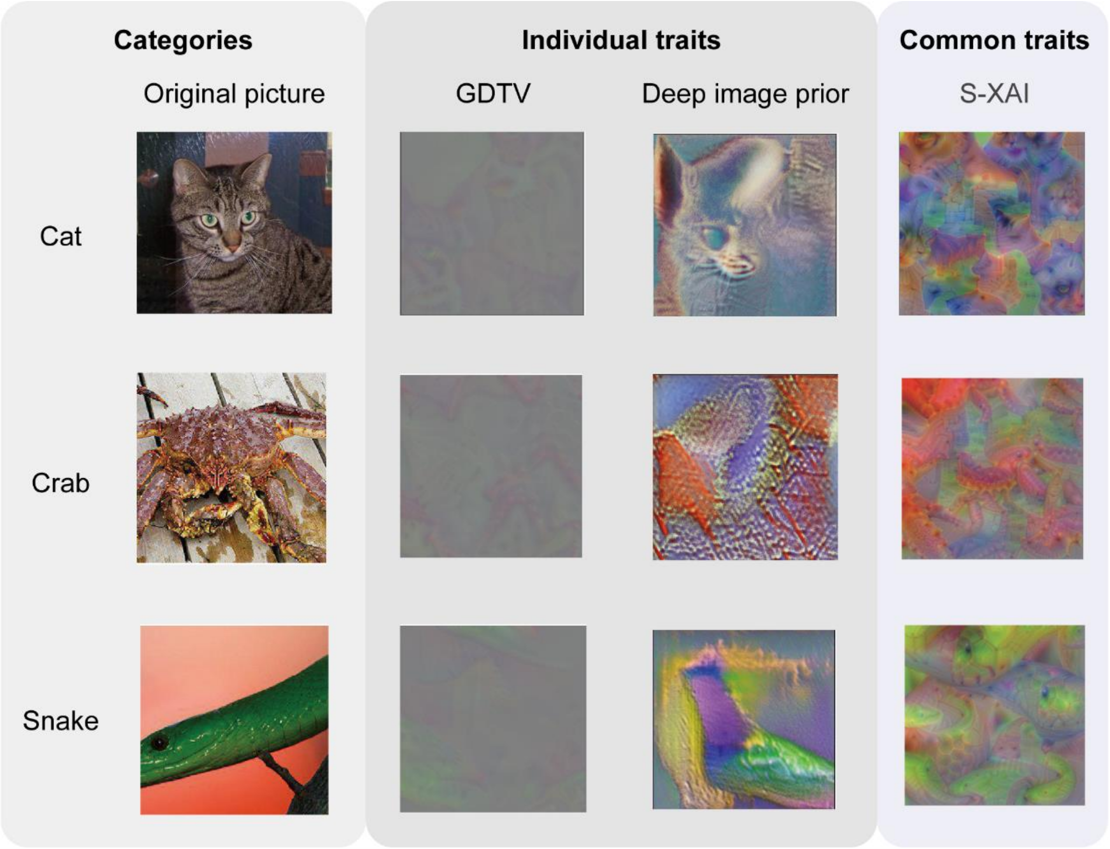

Supplementary Fig. 8. Comparison with existing CNN interpretation methods regarding feature visualization.

References for Supplementary Information

1. Mahendran, A. & Vedaldi, A. Understanding deep image representations by inverting them. in *Proceedings of the IEEE Computer Society Conference on Computer Vision and Pattern Recognition* vols 07-12-June-2015 (2015).
2. Lempitsky, V., Vedaldi, A. & Ulyanov, D. Deep image prior. in *Proceedings of the IEEE Computer Society Conference on Computer Vision and Pattern Recognition* 9446–9454 (2018). doi:10.1109/CVPR.2018.00984.
3. Szegedy, C. *et al.* Intriguing properties of neural networks. in *2nd International Conference on Learning Representations, ICLR 2014 - Conference Track Proceedings* 1–10 (2014).
4. Goodfellow, I. J., Shlens, J. & Szegedy, C. Explaining and harnessing adversarial examples. in *3rd International Conference on Learning Representations, ICLR 2015 - Conference Track Proceedings* (2015).
5. Madry, A., Makelov, A., Schmidt, L., Tsipras, D. & Vladu, A. Towards deep learning models resistant to adversarial attacks. in *6th International Conference on Learning Representations, ICLR 2018 - Conference Track Proceedings* (2018).
6. Chakraborty, A., Alam, M., Dey, V., Chattopadhyay, A. & Mukhopadhyay, D. A survey on adversarial attacks and defences. *CAAI Transactions on Intelligence Technology* vol. 6 25–45 (2021).
7. Meng, D. & Chen, H. MagNet: A Two-Pronged defense against adversarial examples. in *Proceedings of the ACM Conference on Computer and Communications Security* (2017).

doi:10.1145/3133956.3134057.

8. Hendrycks, D. & Gimpel, K. Early methods for detecting adversarial images. in *5th International Conference on Learning Representations, ICLR 2017 - Workshop Track Proceedings* (2019).

9. Metzen, J. H., Genewein, T., Fischer, V. & Bischoff, B. On detecting adversarial perturbations. in *5th International Conference on Learning Representations, ICLR 2017 - Conference Track Proceedings* (2017).

10. Papernot, N., McDaniel, P., Wu, X., Jha, S. & Swami, A. Distillation as a defense to adversarial perturbations against deep neural networks. in *Proceedings - 2016 IEEE Symposium on Security and Privacy, SP 2016* (2016). doi:10.1109/SP.2016.41.
